# Medical Literature

**Published:** 1856-06

**Authors:** 


### MEDICAL LITERATURE.

It will be observed by reading the proceedings of the Am. Med. Association, that reports were presented on the subject embodying somewhat conflicting views in regard to the comparative value of medical productions of this and the old world.

While there can be no doubt but that the claims of medical science on this continent are underrated by our foreign brethren, we perhaps overrate the true estimate. Just claims to much and ingenious originality, unquestionably belong to us in every department of medical science. Indeed we have accomplished as much as could be expected under the means of observation and experiment which are presented to us, and we are not wanting in writers who may justly claim a high character as such. But while this is true, we cannot subscribe to the opinion that, as a whole, our books are equal in practical value or philosophical thought to those of some other countries. We want the patient investigation, the minute enquiry of the German, the systematic and thorough arrangement of the French, the plainess and heavy solidity of our English Calaberators, Rakatansky and Rollecker, Andral, Louis and Valleix Brodie, Churchill, Hall and Sympson have scarcely counterparts on this side the Atlantic. Truly a denial to ourselves the use of such lights as these would dim our medical firmament, whatever nature substitutes might supplant them. The truth is, we need data, comparatively, however well, we might understand how and what to observe in medicine, we generally want the necessary field. Satisfactory establishment of medical facts and principles now-a-days must stand alone on reliable observation. If we theorise we mistake the spirit of the age. Facts observed, arranged, remembered, to be re-affirmed by a repetition of the process establishing their universal existence and unaltering relations and sequences are more and more demanded. When facts are most to be observed, and there are the right sort of men to observe them, we must now look for the productions of medical literature most to be approved, most to be desired. The age of inductive medicine is upon us and we must bow to its requirements. Observe, record, read and reflect, are the admonitions which should ever be sounding in the ear of the medical man. Truth, starting where it may, must ever attract the medical observer. No limit must be assigned his field of vision. We ignore all secularization of science. We most willingly would render homage to him who builds his authority upon the developments of nature in any of the departments of our science, and would ask no questions as to the geography of its origin. Science is untrammelled by lines real or imaginary. Universal in her benefits, she must be universal in her dominions. Let us claim each advance as a victory in which all are interested,—receive as a boon to all, every discovery made or fact established. Emulation is always to be encouraged, and where it results in superior excellency its products may be justly claimed as an honorable distinction to their author and to the country to which he belongs. But sectional strife, which sees little good in aught which emanates beyond a certain boundary, is at all times to be avoided. Our profession, the hand maid of humanity, should be as boundless in her aim, and as universal in her ties. We well know what ignorance is often manifested abroad, of the real state of the science here. But we are also aware of the many and durable contributions to the literature of the profession which we receive from them. The great number and extent of the various hospitals in Europe, as well as the thorough system of medical education, are well calculated to afford the greatest amplitude and readiest facility to medical enquiry, and to place in the way of this enquiry men well prepared for investigation. We have but to glance at the statistical reports upon the various subjects belonging to our profes sion to perceive how vast the field presented for observation in the different hospitals abroad. What lists of cases do we find in every practical treatise! A Boivin or Lachapelle will give you 20,000 cases occurring under their observation in obstetrical practice to illustrate a point, and so of almost every department of medicine. Where so many facts occur, and under the cognisance of such observers, the highest possible value must be awarded their works. It would be endless to enumerate the number of instances which might be adduced to show what great advantages in several particulars, our foreign brethren have over us in this way, and it is by no means derogatory to us that we do not often equal them in illustrative works, on this account.

Where we can convince ourselves that we have a standard work of equal value with an exotic of the best character, we certainly would prefer our own and use our efforts for its adoption; for we would not be understood as wanting in home affection, but as devoid of that local prejudice which obscures the excellencies of foreign productions. But we will go further, and say that the style of our medical works is not, as a general thing, so appropriate to their subjects as some of transatlantic origin. We have usually too much rhetoric, too much display. Our writers too often think too much of how the thing will look upon paper; a matter of fact style is too dull with them for print; the practical is lost in flowers of composition.

But we did not sit down to criticise, so much as to harmonize; not to draw invidious comparisons, so much as oppose secularization of our science; to enter our protest against any measure of preference founded upon other basis than intrinsic worth. When this is equal, our sectional attachments may then turn the scale.

Our duty is to break down rather than erect the walls of separation between the votaries of our noble science. Let all our labors be upon the links of a lengthened, but somewhat dissevered chain, the ultimate aim being its unbroken connection, the result of one grand and comprehensive design; the end, the binding together of all nations, removing the barriers which partial views and prejudices build, and fraternizing us as a common brotherhood, devoted to the perfection of a science, which surrenders to none in the dignity of its pursuits or its close relation to the very well being of the human family.

